# Polymorphisms in *STAT4*, *PTPN2*, *PSORS1C1* and *TRAF3IP2* Genes Are Associated with the Response to TNF Inhibitors in Patients with Rheumatoid Arthritis

**DOI:** 10.1371/journal.pone.0169956

**Published:** 2017-01-20

**Authors:** Paola Conigliaro, Cinzia Ciccacci, Cristina Politi, Paola Triggianese, Sara Rufini, Barbara Kroegler, Carlo Perricone, Andrea Latini, Giuseppe Novelli, Paola Borgiani, Roberto Perricone

**Affiliations:** 1 Clinic of Rheumatology, Allergology and Clinical Immunology, Department of “Medicina dei Sistemi”, University of Rome Tor Vergata, Rome, Italy; 2 Department of Biomedicine and Prevention, Genetics Section, University of Rome Tor Vergata, Rome, Italy; 3 Reumatologia, Dipartimento di Medicina Interna e Specialità Mediche, Sapienza Università di Roma, Rome, Italy; South Texas Veterans Health Care System, UNITED STATES

## Abstract

**Objective:**

Rheumatoid Arthritis (RA) is a progressive autoimmune disease characterized by chronic joint inflammation and structural damage. Remission or at least low disease activity (LDA) represent potentially desirable goals of RA treatment. Single nucleotide polymorphisms (SNPs) in several genes might be useful for prediction of response to therapy. We aimed at exploring 4 SNPs in candidate genes (*STAT4*, *PTPN2*, *PSORS1C1* and *TRAF3IP2*) in order to investigate their potential role in the response to therapy with tumor necrosis factor inhibitors (TNF-i) in RA patients.

**Methods:**

In 171 RA patients we investigated the following SNPs: rs7574865 (*STAT4*), rs2233945 (*PSORS1C1*), rs7234029 (*PTPN2*) and rs33980500 (*TRAF3IP2*). Remission, LDA, and EULAR response were registered at 6 months and 2 years after initiation of first line TNF-i [Adalimumab (ADA) and Etanercept (ETN)].

**Results:**

*STAT4* variant allele was associated with the absence of a good/moderate EULAR response at 2 years of treatment in the whole RA group and in ETN treated patients. The *PTPN2* SNP was associated with no good/moderate EULAR response at 6 months in ADA treated patients. Patients carrying *PSORS1C1* variant allele did not reach LDA at 6 months in both the whole RA group and ETN treated patients. *TRAF3IP2* variant allele was associated with the lack of LDA and remission achievement at 6 months in all RA cohort while an association with no EULAR response at 2 years of treatment occurred only in ETN treated patients.

**Conclusions:**

For the first time, we reported that SNPs in *STAT4*, *PTPN2*, *PSORS1C1*, and *TRAF3IP2* are associated with response to TNF-i treatment in RA patients; however, these findings should be validated in a larger population.

## Introduction

Rheumatoid Arthritis (RA) is a progressive autoimmune disease characterized by chronic joint inflammation and structural damage [1]. The management of RA has undergone significant changes with the current “treat to target” strategy [2]. The introduction of biological disease modifying anti-rheumatic drugs (bDMARDs) has changed the face of RA with remission or at least low disease activity (LDA) as achievable goals [3, 4]. Predictive biomarkers of response to therapy with bDMARDs could enable selection of the optimal treatment for the individual patients. Evidence assessed the value of age, gender, concomitant drugs, body mass index, or smoking status for predicting response to treatment [5–7]. Moreover, RA disease duration, disease activity, functional status, presence of autoantibodies [rheumatoid factor (RF) and anti-citrullinated peptide antibodies (ACPA)], and previous therapies can influence drug response [8–11]. Genetic inter-individual variability can also contribute to the differences in the response to treatment: some single nucleotide polymorphisms (SNPs) showed an association with bDMARDs response and might be useful for prediction, although few associations have been replicated [12–15].

Some genes already known to be involved in RA susceptibility [16] could also be involved in the variability of the response to tumor necrosis factor (TNF)-inhibitors (TNF-i) drugs [15]. Among the known loci associated with RA, the signal transducer and activator of transcription 4 (*STAT4*) could be one of most interesting candidate genes to study in relation to drug response [17]. A recent meta-analysis demonstrated that SNPs in *STAT4* confer susceptibility to RA in total subjects and in major ethnic groups. Moreover, this association was not dependent on RF and ACPA positivity [18]. The protein tyrosine phosphatase non-receptor 2 (*PTPN2*) is one of the newly investigated genes being recently reported as linked to the pathogenesis of RA [19–21]. Additional genetic associations with the development of RA have been suggested for *PSORS1C1/CDSN* and TRAF3 Interacting Protein 2 (*TRAF3IP2*) genes that are well-known susceptibility genes for psoriasis and psoriatic arthritis [21–23].

Thus, the aim of our study was to investigate the potential role of SNPs in *STAT4*, *PTPN2*, *PSORS1C1*, and *TRAF3IP2* as predictors of remission and LDA in a cohort of RA patients treated with first line TNF-i.

## Material and Methods

### Patients

Medical records of RA patients referred to the Rheumatology Outpatient Clinic at the Department of “Medicina dei Sistemi” (“Policlinico Tor Vergata”, Rome, Italy), were retrospectively analyzed (time frame of the enrollment January 2008-December 2013). Patients were included in the study if they fulfilled the following inclusion criteria: the 2010 American College of Rheumatology (ACR)/European League Against Rheumatism (EULAR) classification criteria for RA [24], ≥ 18 years of age, inadequate response to at least one conventional synthetic (cs)DMARD, including Methotrexate, naïve for biologic treatment. Patients were excluded from the study if they showed impairment of hepatic/renal function, alcohol abuse, recent infection (with the last infection >3 month ago), ongoing history of malignancy (with interval malignancy-free >5 years) or ongoing pregnancy, and if they had missing or incomplete data in the follow-up visits. Therefore, the study included 171 RA patients of Caucasian origin. Patients received recommended doses of TNF-i: subcutaneous injection of Adalimumab (ADA) at 40 mg bi-weekly or Etanercept (ETN) at 50 mg every week. Disease activity and clinical response to therapy were assessed using Simplified Disease Activity Index (SDAI; LDA: ≤ 11, remission: ≤ 3.3) [25], disease activity score on 28 joints [DAS28 based on C-reactive protein (CRP)], and EULAR response criteria [25, 26]. The clinical and laboratory findings were evaluated at baseline and every 3 months from the start of TNF-i therapy; data of LDA, remission and EULAR response were registered at 6 months and 2 years after the beginning of the TNF-i treatment. Laboratory assessment included CRP, RF and ACPA. CRP and RF levels were assessed by nephelometry (normal range, 0–3 mg/L and 0–10 IU, respectively). ACPA were detected with a commercial third generation automated chemiluminescent kit: values >20 IU were considered positive. Peripheral blood samples were obtained at the time of the first medical evaluation from all included RA patients in order to perform the genetic analyses. All patients were naïve for biologic treatments at the time of blood sampling. Samples were stored at -80°C until they were analyzed. Written informed consent was obtained from patients. The study protocol was approved by the local ethics committee of the “Policlinico Tor Vergata” in Rome (Italy).

### DNA extraction and genotyping

Genomic DNA was isolated from peripheral blood mononuclear cells using a Qiagen blood DNA mini kit. We have investigated the following SNPs, localized in the genes reported in parenthesis: rs7574865 (*STAT4*), rs7234029 (*PTPN2*), rs2233945 (*PSORS1C1*), and rs33980500 (*TRAF3IP2*). Genotyping was performed by allelic discrimination assay by TaqMan technology (Applied Biosystems, Foster City, CA, USA) and ABI PRISM 7000. Each assay was run including samples with known genotypes.

### Statistical analysis

The Hardy–Weinberg equilibrium was verified for all SNPs by the Pearson χ2 test. We evaluated a possible correlation between the genetic variants and the SDAI LDA, SDAI remission and EULAR response, at 6 months and 2 years from the beginning of the TNF-i treatment. Differences in genotypes frequencies between groups of patients were evaluated by Pearson χ^2^ test or by the Fisher’s Exact test, where appropriate. Odds ratios (ORs) with 95% CI were calculated. A multivariate logistic regression analysis was used to correct the p-value for sex, csDMARDS and ACPA/RF positivity. All statistical analyses were performed by the SPSS program ver. 19 (IBM Corp, Armonk, NY USA). Two-tailed P values less than 0.05 were considered statistically significant.

## Results

A total of 171 RA patients were included in the study, of whom 62.6% (n = 107) were treated with ETN and 37.4% (n = 64) were treated with ADA. Clinical and demographic data of the population are described in Table 1. Patients had longstanding disease in 72.5% of the cases. RF and ACPA were positive in 69.6% and 74.3% of patients, respectively. Mean SDAI at the beginning of the treatment was 27.6 ± 14. Patients with RA receiving concomitant csDMARDs comprised 77.2%. After 6 months of TNF-i treatment, SDAI remission was achieved in 26.7% of the whole RA population, SDAI-LDA was reached in 54% and a good-moderate EULAR response in 73.3% of patients. After 2 years of treatment, SDAI remission was achieved in 29.8%, SDAI-LDA in 63.5%, and a good-moderate EULAR response was reached in 77.9% of patients. At 2 years we observed 65 dropouts patients (38% of the whole study population) because of adverse events (n = 7, 10.7%), secondary failure (n = 33, 50.7%) and concomitant conditions (n = 25, 38.4%). No differences in demographic, clinical data and response to treatment were detected between subgroups of patients treated with ETN or ADA.

**Table 1:** Demographic and clinical data of 171 patients with Rheumatoid Arthritis included in the study.

	Etanercept N = 107	Adalimumab N = 64	All N = 171
Age (years)	54.4 ± 12.8	52 ± 13.5	53.6 ± 13.1
Women, n (%)	80 (74.7)	52 (81.2)	132 (77.2)
Disease duration (years)	11.3 ± 18.2	10.7 ± 19.9	9.2 ± 18.8
Early arthritis (< 2 years), n (%)	31 (28.9)	16 (25)	47 (27.5)
RF positivity, n (%)	75 (70)	44 (68.7)	119 (69.6)
ACPA positivity, n (%)	79 (73.8)	48 (75)	127 (74.3)
Baseline DAS28	5.2 ± 1.3	5.1 ± 1.2	5.2 ± 1.3
Baseline SDAI	27.6 ± 14	26.5 ± 12.9	27.2 ± 13.6
Concurrent csDMARDs, n (%)	78 (72.9)	50 (78.1)	132 (77.2)
Concurrent PDN, n (%)	54 (50.4)	33 (51.5)	87 (50.9)

Data presented as number of patients (%) or mean ± SD. RF, Rheumatoid Factor; ACPA, anti-citrullinated peptide antibodies; DAS28, Disease Activity Score on 28 joints; SDAI, Simplified Disease Activity Index; csDMARDs, conventional synthetic disease-modifying antirheumatic drugs; PDN, prednisone.

### Associations of genetic variants with response to TNF-i treatment

We analyzed four SNPs in four candidate genes to investigate their possible role on TNF-i treatment response. In particular, we compared the genotypes distribution in relation to SDAI LDA (achieved *vs* not achieved), SDAI remission (achieved *vs* not achieved), and EULAR response (good/moderate *vs* no response). All the analyses were performed considering the clinical evaluations during follow up at 6 months and at 2 years after the treatment starting. Firstly, we performed a primary analysis considering the whole cohort of RA patients, independently from the specific administered TNF-i drug (Table 2). In accordance with this preliminary analysis the *TRAF3IP2* SNP was associated with no achievement of LDA and remission at 6 months (P = 0.035 and OR = 0.36, P = 0.013 and OR = 0.11, respectively). These associations were confirmed after multiple correction for sex, csDMARDS and ACPA/RF positivity (P_adj_ = 0.03 and P_adj_ = 0.02 respectively). *STAT4* SNP was associated with no EULAR response at 2 years of treatment (P = 0.05, OR = 0.38). Patients carrying *PSORS1C1* variant allele did not reach LDA at 6 months (P = 0.002, OR = 0.35). The association with lack of achievement of LDA at 6 months was confirmed by the multiple correction (P_adj_ = 0.003, OR_adj_ = 0.36).

**Table 2:** Association analysis between TRAF3IP2, STAT4, PSORS1C1 and PTPN2 polymorphisms and response to TNF-inhibitors treatment in RA patients.

SNP and gene	Target	Response	Six months					Two years				
			Genotypes wt/hz/var	P (1dl)	OR (95% CI)	P_adj_*	OR_adj_ (95% CI)	Genotypes wt/hz/var	P (1dl)	OR (95% CI)	P_adj_*	OR_adj_ (95% CI)
rs33980500 C>T *TRAF3IP2*	SDAI_LDA	Yes	81/7/0	**0.035**	0.36 (0.14–0.96)	**0.03**	0.33 (0.12–0.9)	61/6/0	0.31	0.54 (0.16–1.81)	0.31	0.53 (0.16–1.79)
		No	59/13/1					33/5/1				
	SDAI_remission	Yes	43/1/0	**0.013**	0.1 (0.02–0.87)	**0.02**	0.09 (0.01–0.70)	31/1/0	0.077	0.18 (0.02–1.47)	0.11	0.18 (0.02–1.49)
		No	97/19/1					62/10/1				
	EULAR	Good/moderate	103/14/1	0.60	0.77(0.29–2.04)	0.45	0.68 (0.25–1.87)	74/7/1	0.31	0.51 (0.14–1.89)	0.32	0.51 (0.14–1.91)
		No	37/7/0					19/4/0				
rs7574865 G>T *STAT4*	SDAI_LDA	Yes	49/34/5	0.58	0.84 (0.45–1.56)	0.54	0.82 (0.44–1.54)	34/27/6	0.65	0.83 (0.38–1.84)	0.6	0.81 (0.36–1.79)
		No	38/32/4					18/18/3				
	SDAI_remission	Yes	25/15/4	0.63	0.84 (0.42–1.69)	0.68	0.86 (0.42–1.75)	12/18/2	0.13	1.91 (0.82–4.48)	0.12	2 (0.84–4.76)
		No	62/51/5					39/27/7				
	EULAR	Good/moderate	67/45/7	0.22	0.65 (0.32–1.3)	0.22	0.64 (0.31–1.3)	44/31/7	**0.05**	0.38 (0.14–1.02)	0.06	0.38 (0.14–1.03)
		No	20/22/2					7/14/2				
rs2233945 C>A *PSORS1C1*	SDAI_LDA	Yes	65/21/2	**0.002**	0.35 (0.18–0.68)	**0.003**	0.36 (0.19–0.71)	41/25/1	0.82	0.91 (0.41–2.04)	0.85	0.92 (0.41–2.08)
		No	37/33/4					23/13/3				
	SDAI_remission	Yes	31/11/2	0.23	0.63 (0.30–1.34)	0.29	0.66 (0.31–1.41)	23/8/1	0.13	0.5 (0.2–1.23)	0.12	0.49 (0.2–1.21)
		No	71/43/4					41/29/3				
	EULAR	Good/moderate	79/37/3	0.16	0.61 (0.30–1.23)	0.24	0.65 (0.32–1.33)	46/34/2	**0.05**	2.82 (0.95–8.32)	0.06	2.9 (0.97–8.67)
		No	24/17/3					18/3/2				
rs7234029 A>G *PTPN2*	SDAI_LDA	Yes	64/22/2	0.31	0.71 (0.36–1.38)	0.25	0.66 (0.33–1.33)	46/20/1	0.23	1.77 (0.7–4.5)	0.24	1.77 (0.69–4.58)
		No	47/23/2					31/7/1				
	SDAI_remission	Yes	32/10/2	0.57	0.80 (0.37–1.73)	0.42	0.72 (0.33–1.59)	23/8/1	0.94	1.04 (0.41–2.62)	0.93	1.05 (0.40–2.71)
		No	79/35/2					53/19/1				
	EULAR	Good/moderate	86/29/3	0.11	0.55 (0.26–1.14)	0.08	0.5 (0.23–1.08)	59/22/1	0.85	1.11 (0.39–3.15)	0.77	1.17 (0.40–3.44)
		No	25/16/1					17/5/1				

wt" indicates the homoziygous genotype for the wild-type allele; "hz" indicates the heterozygous genotype; "var" indicates the homozygous genotype for the variant allele.

* P adjusted for sex, DMARDS and ACPA/RF positivity. Significant P values are reported in bold.

In a second step, we repeated the analysis with each drug considered separately and we observed that the associations were drug-specific, except for the *TRAF3IP2* SNP (Table 3 and Table 4). Indeed, TRAF3IP2 SNP showed a trend for association with lack of achievement of remission in ETN and ADA treated patients at 6 months of treatment after adjusting for sex, csDMARDS and ACPA/RF positivity (ETN: P_adj_ = 0.08, OR_adj_ = 0.14; ADA: P_adj_ = 0.09, OR_adj_ = 0.14).

**Table 3:** Association between analyzed polymorphisms and response to Etanercept treatment in RA patients.

SNP and gene	Target	ETN Response	Six Months						Two years				
			Genotypes wt/hz/var		P (1dl)	OR (95% CI)	_adj_*	OR_adj_ (95% CI)	Genotypes wt/hz/var	P (1dl)	OR (95% CI)	P_adj_*	OR_adj_ (95% CI)
rs33980500 C>T *TRAF3IP2*	SDAI_LDA	Yes	45/4/0	0.15		0.41 (0.12–1.42)	0.13	0.36 (0.1–1.36)	41/3/0	0.56	0.61 (0.11–3.26)	0.61	0.64 (0.12–3.51)
		No	41/8/1						25/2/1				
	SDAI_remission	Yes	28/1/0	0.066		0.17 (0.02–1.40)	0.08	0.14 (0.02–1.23)	21/1/0	0.43	0.42 (0.05–3.82)	0.56	0.51 (0.05–4.98)
		No	58/11/1						44/4/1				
	EULAR	Good/moderate	60/8/1	0.68		0.78 (0.24–2.55)	0.75	0.88 (0.23–2.89)	52/5/1	0.23	ND	1	ND
		No	26/5/0						13/0/0				
rs7574865 G>T *STAT4*	SDAI_LDA	Yes	23/23/3	0.84		1.09 (0.5–2.38)	0.82	1.1 (0.48–2.50)	21/19/4	0.91	0.95 (0.37–2.45)	0.8	0.88 (0.33–2.33)
		No	25/22/4						13/12/3				
	SDAI_remission	Yes	15/11/3	0.63		0.81 (0.34–1.93)	0.7	0.84 (0.34–2.06)	8/14/0	0.25	1.82 (0.65–5.12)	0.2	2.08 (0.68–6.34)
		No	33/34/4						25/17/7				
	EULAR	Good/moderate	34/31/5	0.75		0.87 (0.37–2.04)	0.78	0.88 (0.36–2.17)	31/22/5	**0.013**	0.16 (0.03–0.78)	**0.02**	0.14 (0.03–0.73)
		No	14/15/2						2/9/2				
rs2233945 C>A *PSORS1C1*	SDAI_LDA	Yes	36/12/1	**0.012**		0.35 (0.15–0.80)	**0.023**	0.37 (0.16–0.87)	24/19/1	0.94	0.96 (0.37–2.49)	0.9	1.07 (0.40–2.83)
		No	25/24/2						15/11/2				
	SDAI_remission	Yes	20/8/1	0.3		0.62 (0.25–1.54)	0.38	0.66 (0.26–1.67)	13/8/1	0.64	0.78 (0.28–2.17)	0.64	0.77 (0.26–2.29)
		No	41/28/2						26/21/2				
	EULAR	Good/moderate	47/22/1	0.074		0.46 (0.19–1.09)	0.11	0.48 (0.2–1.18)	30/26/2	0.25	2.1 (0.58–7.59)	0.21	2.35 (0.63–8.84)
		No	15/14/2						9/3/1				
rs7234029 A>G *PTPN2*	SDAI_LDA	Yes	34/14/1	0.39		0.7 (0.30–1.61)	0.33	0.64 (0.26–1.56)	32/12/0	0.83	1.13 (0.38–3.32)	0.77	1.19 (0.39–3.65)
		No	30/19/0						21/7/0				
	SDAI_remission	Yes	18/10/1	0.66		1.22 (0.5–3.01)	0.74	1.17 (0.46–3.01)	15/7/0	0.52	1.44 (0.48–4.36)	0.52	1.49 (0.44–5.03)
		No	46/23/0						37/12/0				
	EULAR	Good/moderate	46/22/1	0.66		0.82 (0.33–2.02)	0.77	0.86 (0.33–2.89)	43/15/0	0.72	0.79 (0.21–2.93)	0.86	0.88 (0.22–3.5)
		No	18/11/0						9/4/0				

"wt" indicates the homoziygous genotype for the wild-type allele; "hz" indicates the heterozygous genotype; "var" indicates the homozygous genotype for the variant allele.

* P adjusted for sex, DMARDS and ACPA/RF positivity. Significant P values are reported in bold.

**Table 4:** Association between analysed polymorphisms and response to Adalimumab treatment in RA patients.

SNP and gene	Target	ADA response	Six months					Two years				
			Genotypes wt/hz/var	P (1dl)	OR (95% CI)	P_adj_*	OR_adj_ (95% CI)	Genotypes wt/hz/var	P (1dl)	OR (95% CI)	P_adj_*	OR_adj_ (95% CI)
rs33980500 C>T *TRAF3IP2*	SDAI_LDA	Yes	36/3/0	0.11	0.3 (0.06–1.4)	0.11	0.25 (0.05–1.37)	20/3/0	0.31	0.4 0.07–2.42)	0.37	0.41 (0.06–2.87)
		No	18/5/0					8/3/0				
	SDAI_remission	Yes	15/0/0	0.09	ND	1	ND	10/0/0	0.08	ND	1	ND
		No	39/8/0					18/6/0				
	EULAR	Good/moderate	43/6/0	0.76	0.77 (0.14–4.34)	0.73	0.71 (0.10–4.86)	22/2/0	**0.027**	0.14 (0.02–0.93)	0.16	0.16 (0.02–1.57)
		No	11/2/0					6/4/0				
rs7574865 G>T *STAT4*	SDAI_LDA	Yes	26/11/2	0.42	0.65 (0.23–1.88)	0.46	0.66 (0.22–1.98)	13/8/2	0.55	0.64 (0.15–2.72)	0.48	0.58 (0.13–2.61)
		No	13/10/0					5/6/0				
	SDAI_remission	Yes	10/4/1	0.73	0.81 (0.24–2.74)	0.58	0.7 (0.2–2.46)	4/4/2	0.33	2.1 (0.47–9.44)	0.37	2.12 (0.42–10.81)
		No	29/17/1					14/10/0				
	EULAR	Good/moderate	33/14/2	0.16	0.42 (0.12–1.44)	0.16	0.39 (0.11–1.45)	13/9/2	0.82	0.85 (0.19–3.71)	0.53	0.58 (0.11–3.16)
		No	6/7/0					5/5/0				
rs2233945 C>A *PSORS1C1*	SDAI_LDA	Yes	29/9/1	0.075	0.38 (0.13–1.12)	0.073	0.35 (0.11–1.10)	17/6/0	0.94	0.94 (0.19–4.76)	0.88	0.88 (0.16–4.8)
		No	12/9/2					8/2/1				
	SDAI_remission	Yes	11/3/1	0.5	0.64 (0.18–2.33)	0.43	0.58 (0.15–2.2)	10/0/0	**0.024**	ND	1	ND
		No	30/15/2					15/8/1				
	EULAR	Good/moderate	32/15/2	0.79	1.20 (0.32–4.46)	0.75	1.25 (0.32–4.85)	16/8/0	0.16	4.5 (0.48–42)	0.23	4.24 (0.40–44.55)
		No	9/3/1					9/0/1				
rs7234029 A>G *PTPN2*	SDAI_LDA	Yes	30/8/1	0.79	0.85 (0.26–2.8)	0.87	0.91 (0.27–3.04)	14/8/1	0.072	6.43 (0.7–59.17)	0.09	6.94 (0.73–65.68)
		No	17/4/2					10/0/1				
	SDAI_remission	Yes	14/0/1	0.069	0.17 (0.02–1.41)	0.09	0.15 (0.02–1.32)	8/1/1	0.44	0.5 (0.09–2.93)	0.4	0.43 (0.06–2.99)
		No	33/12/2					16/7/1				
	EULAR	Good/moderate	40/7/2	**0.038**	0.26 (0.07–0.97)	**0.06**	0.27 (0.07–1.03)	16/7/1	0.44	2 (0.34–11.70)	0.37	2.43 (0.35–17.08)
		No	7/5/1					8/1/1				

"hz" indicates the heterozygous genotype; "var" indicates the homozygous genotype for the variant allele.

* P adjusted for sex, DMARDS, ACPA/RF positivity. Significant P values are reported in bold. Between analysed polymorphisms and response to Adalimumab treatment in RA patients.

With regard to ETN treatment, we confirmed an association between EULAR response and *STAT4* SNP: patients carrying the variant allele showed a worse EULAR response at 2 years with P = 0.013 and OR = 0.16 (confirmed also after correction for sex, DMARDS and ACPA/RF positivity, P_adj_ = 0.02 and OR_adj_ = 0.14). The lack of achievement of LDA was also observed for patients carrying the variant allele of *PSORS1C1* SNP (P = 0.012, OR = 0.35; after correction P_adj_ = 0.023, OR_adj_ = 0.37). Regarding the ADA treatment, we observed an association between EULAR response and *TRAF3IP2* SNP at 2 years of treatment (P = 0.027, OR = 0.14): patients carrying the variant allele had a worse response to treatment. The *PSORS1C1* SNP was associated with SDAI remission (P = 0.024) at 2 years of treatment, but the association was not confirmed after correction. Lastly, the *PTPN2* SNP resulted associated with a worse EULAR response at 6 months of ADA treatment (P = 0.038, OR = 0.26), data partially confirmed also after multiple correction (P_adj_ = 0.06, OR_adj_ = 0.27).

The association of *PSORS1C1* with a good-moderate EULAR response, detected in the preliminary analysis in the whole cohort, was not confirmed considering each drug separately. Nonetheless, it is worth to highlight that genotypes carrying the variant allele presented an higher frequency in patients with a good/moderate response at 2 years with both drugs (OR_adj_ = 2.35 in the case of ETN; OR_adj_ = 4.24 in the case of ADA), even if this difference did not reach the statistical significance (P_adj_ = 0.21 and P_adj_ = 0.23 respectively).

**Fig 1:**
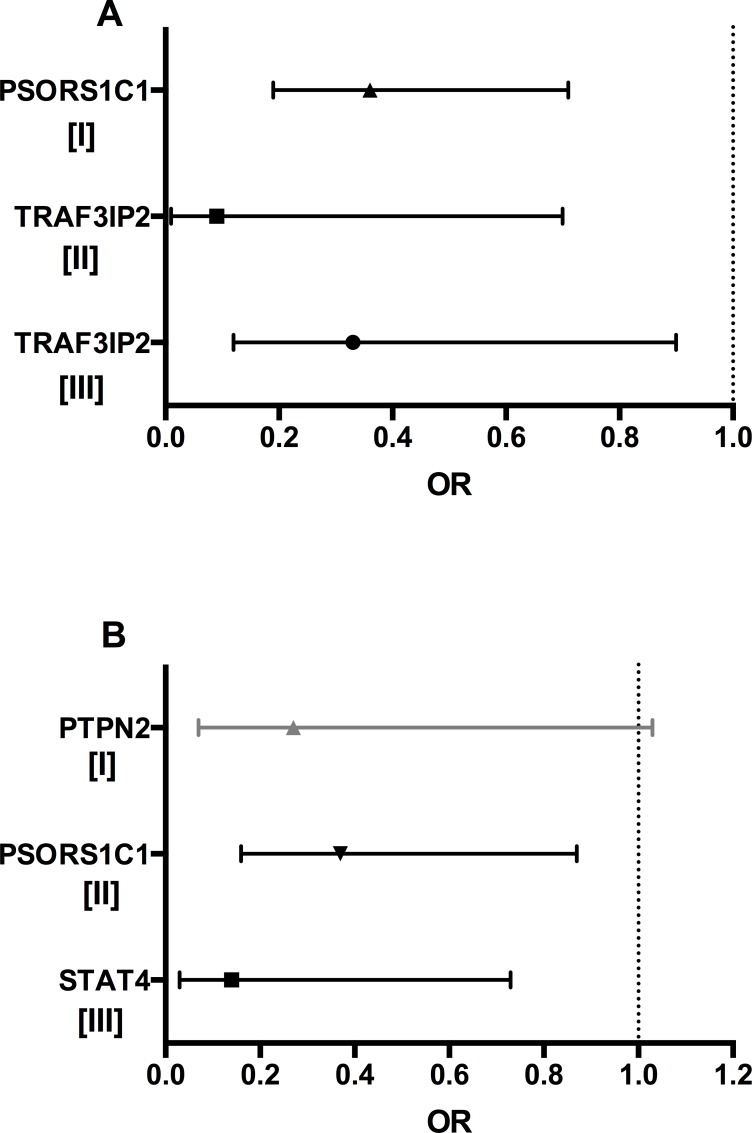
Odds ratio for associations between genetic variants and response to TNF-i treatment. (**A**) Associations in the whole cohort of Rheumatoid Arthritis patients at 6 months of treatment. [I] SDAI LDA (p 0.003); [II] SDAI remission (p 0.02); [III] SDAI LDA (p 0.03). (**B)** Associations in patients treated with Adalimumab (grey line) and Etanercept (black lines) considered separately. [I] EULAR response at 6 months of follow up (p 0.06); [II] SDAI LDA at 6 months of follow up (p 0.023,); [III] EULAR response at 2 years of follow up (p 0.02). Multivariate logistic regression analysis was used to correct the p-value for sex, csDMARDS and ACPA/RF positivity. Odds ratios (OR) with 95% CI were reported.

Fig 1 summarizes the odds ratio of the relevant associations observed between genetic variants and treatment outcome both in the whole RA population and in the two subgroups stratified by the drug (Fig 1).

## Discussion

TNF-i have shown a good efficacy in the treatment of chronic inflammatory arthropathies, including RA [27]. However, a consistent part of patients does not reach the therapeutic targets of remission and LDA during TNF-i treatment. Evidence suggested the presence of a confined period of time, defined “window of opportunity”, in which RA patients are more susceptible to treatment [28, 29]. Moreover, a prolonged disease activity and a long disease duration at treatment initiation have been associated with unfavorable outcomes in RA [29, 30]. Furthermore, RA can cause progressive disability that leads to high direct and indirect costs for the health system [31]. Therefore, the identification of specific predicting factors of response to a specific treatment would be enormously useful in the clinical practice to select the patient that would benefit or not from the treatment [27, 32]. Candidate genes encoding proteins involved in the immune response have been fairly investigated to search for a possible association with TNF-i response in several autoimmune diseases, including RA [33–36]. However, validated genomic biomarkers currently do not significantly allow the identification of non-responders before treatment in RA [15].

In this study, we evaluated the potential role of SNPs in *STAT4*, *PTPN2*, *PSORS1C1*, and *TRAF3IP2* genes on the response to ETN and ADA treatment in RA patients. We selected these genes on the base of our previous studies that aimed to verify the association of common variants with different autoimmune diseases susceptibility [21, 37–39]. In our previous study, *STAT4*, one of the most associated gene with RA susceptibility, related with a higher susceptibility to develop RA and with ACPA positivity, while SNPs in *PSORS1C1* and *PTPN2* genes were differently associated with joint damage in RA, even if we did not observe an association with RA susceptibility [21]. We considered as a candidate gene also *TRAF3IP2* that encodes for Act1, an IL-17R adaptor protein sharing intracellular signal transduction molecules with the TNF-α signaling pathway, serving both as negative regulator of adaptive immunity and as a positive signaling adaptor in IL17-mediated immune responses [40, 41].

The results of the present study showed the presence of some associations of the investigated SNPs with remission, LDA, and EULAR response. Several lines of evidence in the literature demonstrated that ADA and ETN exert a different effect on innate and adaptive immune cell population in RA treated patients despite their similar effectiveness [42–44]. Our preliminary data seems to suggest that the associations may be drug specific. However, given the small number of analyzed subjects, these results could be attributable to a low power of the study and need to be further investigated in larger samples. Regarding the response to ETN treatment, we observed a worse EULAR response in patients carrying the variant allele of *STAT4* rs7574865 after 2 years of treatment and a lack of LDA achievement at 6 months of treatment in patients carrying the variant allele of *PSORS1C1* rs2233945 SNP. These two associations were not present in the group of patients treated with ADA in which, on the contrary, we observed a worse EULAR response in patients carrying the variant allele of *TRAF3IP2* after 2 years of treatment and the variant allele of *PTPN2* after 6 months of treatment.

A recent study reported that the T allele of rs7574865 is significantly associated with higher levels of *STAT4* mRNA and protein expression in a population of patients with early arthritis [45]. Since *STAT4* is involved in the signaling of IL-12, IL-23, and IFN-γ, it has been suggested that patients carrying the rs7574865 minor allele might show stronger T helper (Th)1 and Th17 cytokine responses that are represented in RA [46–48]. ETN seems able to downregulate both the Th1 and Th17 [49], therefore we could hypothesize that patients carrying the variant allele are less sensitive to the ETN effect. On the contrary, we could not demonstrate an association with ADA treatment that might be explained by previous findings suggesting that ADA seems to increase *STAT4* activation in CD4+ T cells from RA patients [50].

We found that the variant allele of *PTPN2* (rs7234029) was associated with a worse EULAR response at 6 months of ADA treatment. The same polymorphism, located in an intron region, was associated with a poor prognosis in a Portuguese population of RA patients treated with DMARDs and biologics [51]. *PTPN2* was significantly overexpressed in synovial tissue samples from RA patients [52]. Interestingly, we observed a high frequency of bone erosions in patients carrying the variant allele [21]; this could be congruent with the worse response to treatment in patients carrying such variant. Although there are no functional studies on rs7234029 SNP, an *in silico* analysis revealed that it modulates potentially the binding sites of several transcription factors involved in inflammation [53]. Therefore, further analysis should firstly replicate our preliminary data on large cohort of patients and then define the potential effect of this SNP on different drug’s mechanism of action.

*PSORS1C1* can affect IL-17 secretion that plays important roles in synovial inflammation and bone destruction in RA [54]. A significant increase in expression of PSORS1C1 in RA synovial tissues has been also described [55]. The functional role of rs2233945 is not known; the SNP is located in an intronic region and therefore we can speculate that it could be involved in expression regulation. Patients carrying the variant allele of this SNP treated with ETN are less likely to achieve LDA at 6 months suggesting that this genetic variation may interfere with the drug mechanism of action possibly increasing the IL-17 response. However, an opposite effect was suggested at 2 years of treatment in the whole RA population and in particular in the ADA group of patients. This contradictory result may be attributed to either a different mechanism of action of ADA in those patients carrying the variant allele, or to a bias related to the small number of analyzed patients and the dropouts at 2 years. Functional studies on this SNP will give important information on its putative role in TNF-i treated patients.

Regarding *TRAF3IP2*, we observed that remission and LDA at 6 months of treatment were not achieved in patients carrying the variant genotype, considering patients treated with ETN and ADA together, but this result was not replicated in the two subgroups stratified by the drug. *TRAF3IP2* product interacts with TRAF proteins; in the *TRAF* gene family, *TRAF1* is a negative regulator of TNF receptor that was identified as a risk locus for RA in a GWAS [56]. The rs33980500 SNP decreases the binding with TRAF6 and this could also alter TRAF2 and TRAF5 protein interactions within the IL-17R signaling pathway, leading to increased neutrophil chemotaxis and an enhanced immune response [57]. Recently, *TRAF1* has been investigated in relation to TNF-i response by Canhao et al., who reported in Portuguese RA patients an association of the minor (G) allele of rs3761847 in the *TRAF1/C5* with a poor response to TNF-i treatment at 6 months [58]. However, another study in a Greek population did not confirm the association of *TRAF1* with TNF-i treatment [59].

This study presents some limitations. First, we have analyzed only one SNP for each selected gene. Indeed, it is possible that other SNPs (or a combination of SNPs) in these genes (and in other genes) could play a role in response to TNF-i treatment. Moreover, the small number of investigated patients may represent a bias that can confound the interpretation of the results, especially in the analysis concerning the two subgroups stratified by the drug. Given the limited number of patients, we have not performed the multiple comparisons correction, and therefore our results should be considered as preliminary data. Further studies are necessary to replicate our findings in a larger cohort of RA patients together with functional studies in order to confirm and better explore the contribution of these SNPs in the treatment response.
